# A Geospatial Modelling Approach Integrating Archaeobotany and Genetics to Trace the Origin and Dispersal of Domesticated Plants

**DOI:** 10.1371/journal.pone.0012060

**Published:** 2010-08-11

**Authors:** Jacob van Etten, Robert J. Hijmans

**Affiliations:** 1 International Rice Research Institute, Los Baños, Philippines; 2 IE University, Segovia and Madrid, Spain; 3 Department of Environmental Science and Policy, University of California Davis, Davis, California, United States of America; University College London, United Kingdom

## Abstract

**Background:**

The study of the prehistoric origins and dispersal routes of domesticated plants is often based on the analysis of either archaeobotanical or genetic data. As more data become available, spatially explicit models of crop dispersal can be used to combine different types of evidence.

**Methodology/Principal Findings:**

We present a model in which a crop disperses through a landscape that is represented by a conductance matrix. From this matrix, we derive least-cost distances from the geographical origin of the crop and use these to predict the age of archaeological crop remains and the heterozygosity of crop populations. We use measures of the overlap and divergence of dispersal trajectories to predict genetic similarity between crop populations. The conductance matrix is constructed from environmental variables using a number of parameters. Model parameters are determined with multiple-criteria optimization, simultaneously fitting the archaeobotanical and genetic data. The consilience reached by the model is the extent to which it converges around solutions optimal for both archaeobotanical and genetic data. We apply the modelling approach to the dispersal of maize in the Americas.

**Conclusions/Significance:**

The approach makes possible the integrative inference of crop dispersal processes, while controlling model complexity and computational requirements.

## Introduction

Understanding the domestication and the subsequent dispersal of cultivated plants is fundamental to our comprehension of the rise of early agricultural societies [1]–[2]. Such insights may also have much practical relevance. The initial dispersal of crops is an important determinant of the geographic distribution of their genetic diversity. A better understanding of this distribution can help assist in establishing representative collections of crop genetic resources, which form the basis of modern crop improvement [3]–[4].

Research on plant domestication and crop dispersal is a multidisciplinary effort with the principal contributions coming from archaeology and molecular biology [5]–[6]. The application of new research techniques has led to the availability of significant amounts of new data. Retrieval of archaeobotanical remains by flotation, introduced in the 1960s [7], made it possible to systematically collect data across archaeological sediments and sites. Accelerator mass spectrometry (AMS) radiocarbon dating has been used to accurately determine the age of microscopic archaeological remains [8], while scanning electron microscopy (SEM) has improved the taxonomic identification of archaeobotanical remains. The retrieval of phytoliths and starch has also provided new opportunities for data collection [9]. DNA analysis, applied to both archaeological crop remains and traditional crop varieties, has helped to elucidate evolutionary relationships and trajectories [10]–[11].

In spite of this increase in the availability and quality of data, conflicting views persist regarding the evolutionary and geographical trajectories of crops. The issue does not seem to be data availability alone. For instance, for Asian rice (*Oryza sativa* L.), much genetic and archaeological data are available, yet there is no consensus regarding where it was domesticated and how it spread across the Asian continent [12]–[13]. Sometimes, radical conclusions are drawn from a few new data points without considering the full body of evidence [14]. Therefore, not only more data are needed, but also better and different ways to analyze the data in a comprehensive way. Since crop evolution involves processes at multiple levels of biological organization, integrative approaches that link these levels are needed [15]. Linking genes, crops, and landscapes through a geographical analysis of genetic and archaeobotanical data is one important way to achieve such multilevel integration [6]. Geospatial models have an important role to play in this.

Spatially explicit models of dispersal are not new to archaeology. Models of diffusion have been used to represent the spread of Neolithic innovations, including pottery, copper metallurgy, cultivated maize [16], and the Neolithic emergence of agriculture in Europe [17]–[20]. These relatively simple models show a good correspondence with the spatial pattern of radiocarbon-dated archaeological evidence and what is known about the origins of agriculture. Also dynamic simulation has been applied to crop dispersal [21].

In contrast, current methods used for the geographical analysis of crop genetic diversity are generally not spatially explicit. A common approach to determine the geographical origin of a crop is to locate the genetically closest wild progenitor population [e.g.], [ 22]–[24]. This type of evidence, although very important, may not always be conclusive. The ancestral population may have shifted geographically, it may have become extinct over time, or it may have been omitted during sampling. The spatial pattern of intraspecies crop genetic diversity provides independent information about the origin of the crop as well as information about the dispersal routes followed. However, spatial analysis of crop genetic data is currently mostly done *post hoc*, for instance, by determining genetic clusters and plotting these on maps. A spatially explicit model would be needed to integrate archaeobotanical and genetic evidence of crop origins and dispersal routes.

In the broader field of phylogeography, the need for further integration of genetics and geography is increasingly recognized [25]. Recently, visualization and inference methods based on phylogenetic trees have been developed [26]–[28]. Progress has also been made by combining niche models and coalescent models [29] and modelling gene flow as random walks in heterogeneous landscapes [30]. Range expansion, of specific interest here, has been modelled with spatially explicit simulation [31] and analyzed by comparing genetic and geographic distances along dispersal routes [32]. Such methods have not yet been applied to cultivated plants, however.

We present a novel, integrated approach, which combines different elements from existing archaeological and genetic geospatial models. Our approach has at its core a landscape model that represents the ease of movement through geographical space. Given the geographical origin of a crop, we derive from the landscape model different distance measures that can be quantitatively related to (1) the radiocarbon dates for the first appearance of the crop in the archaeological record, (2) heterozygosity of (contemporary) crop samples, and (3) genetic distances between these samples. The measures are all based on (randomized) shortest path metrics that can be obtained without stochastic simulation. This provides greater computational speed, allowing for the evaluation of alternative locations of crop origins and of different variables of potential influence on crop dispersal.

We apply the approach to maize dispersal in the Americas. Although there is still debate about the exact geographical origin of maize, it is thought to lie in a limited area in southern Mexico, where its closest wild relatives occur naturally [33]. The spread beyond this area was exclusively the result of human action. Introgression from wild *Zea mays* subspecies into cultivated maize is “measurable but modest” [22], [34]. *Zea mays* ssp. *mexicana* contributed an estimated 2.3% of the genomes of maize samples sympatric to this subspecies [22]. The most probable scenario for introgression from *Zea mays* ssp. *parviglumis* is that it took place early after divergence and was followed by the gradual isolation of the taxa [34]. If maize spread out of Mesoamerica after isolation was completed, this influence is not important. Because of its single origin and limited geneflow with wild relatives, maize provides a relatively uncomplicated case compared with other crops and is therefore particularly suited to evaluate our approach. We performed a single modelling iteration with a simple model to demonstrate and evaluate our new modelling approach. In the final section, we discuss next modelling iterations and possible extensions of the approach.

## Methods

### General description of the modelling approach

#### Landscape model

The initial dispersal of crops is affected by several geographical factors such as the location of water bodies, environmental barriers, the suitability of environments to grow the crop, and prehistoric human population density. To model the relative influence of different factors, we use conductance matrices derived from gridded geographic data. In the conductance matrix, each grid cell is represented by a row with values indicating the conductance or relative ease of crop dispersal and gene flow to other cells on the grid. A grid with n cells produces an n×n cells conductance matrix. Generally, we connect pairs of spatially adjacent cells, which receive non-zero values in the conductance matrix, while unconnected pairs of cells receive a zero. Spatially non-adjacent cells could be connected in the conductance matrix to represent long-distance ‘leap-frog’ movements. Here, to keep the model simple and following a number of existing models in archaeology [18]–[20] and spatial genetics [30]–[31], we connect spatially adjacent cells only.

What we call a “conductance matrix” is called a “weighted adjacency matrix” in graph theory. In this context, however, we prefer the term “conductance matrix” to avoid confusion, as adjacency between nodes in the graph derived from the grid does not necessarily imply *spatial* adjacency between the cells represented by the nodes. A further reason for this terminology is that the ease of transition can be seen as equivalent to conductance in electrical terms [30], [35]–[36]. Grid cells represent the nodes of a mesh, each connected to its neighbors by resistors. Conductance is the reciprocal of resistance (conductance = 1/resistance), which in turn is equivalent to friction or cost, which are terms more commonly used in geospatial analysis.

Using conductance matrices has a number of advantages. In geospatial analysis, least-cost distances are generally calculated from a cost or friction grid [37]. However, a conductance matrix is more versatile as it can represent connections between non-adjacent cells and anisotropy (e.g., the friction from cell i to j being unequal to the friction from j to i). Also, a conductance matrix can be used directly to derive distance metrics based on random walks (see below). Conductance matrices generally contain a large number of zeros and few non-zero values. Hence, conductance matrices can be handled as *sparse* matrices because most values are zero. Sparse matrices only store (indexed) non-zero values, which is very efficient memory-wise. Also, fast computational methods are available for sparse matrices.

Conductance values are determined from the values of the two grid cells that are connected, using different functions. Simple functions, such as the average, or functions that require parameters can be used. The conductance values need to be corrected for (1) differences between diagonal and non-diagonal connections between cells if cells are connected in more than four directions and (2) distance distortions, specifically the decreasing W-E distance between cell centres on a longitude-latitude grid when moving from the equator towards the poles. Both issues are addressed by dividing conductance values by the distances between the cell centres.

We refer to the final conductance matrix used to calculate the distance metrics as the *landscape model* (note that the term ‘landscape’ does not imply a certain range of geographical extent or resolution; our landscape model can cover an entire continent). The landscape model is constructed by combining various conductance matrices for the selected variables that might influence crop dispersal. Conductance matrices based on different variables are combined into a single conductance matrix (giving each variable a certain weight), which forms our landscape model. The complexity of the landscape model can vary, depending on the number of conductance matrices (i.e., weight parameters) and the number of parameters required by the function(s) used to determine the values in each of the conductance matrices.

Given a certain geographical origin of crop dispersal and a landscape model, we can predict the movement of crops in geographical space and, consequently, the age of archaeobotanical crop remains, heterozygosity, and genetic distances between crop populations. The following sections discuss the construction of predictor variables from the landscape model and the model-fitting procedure. The modelling approach requires measures of goodness-of-fit between the landscape model on the one hand and the archaeobotanical and genetic data on the other. To derive these measures, we use and adapt elements of existing modelling approaches in both archaeology and geographical genetics.

#### Modelling crop remain radiocarbon ages

We use a variant of existing spatial diffusion models in archaeology for the post-domestication diffusion of crops [16]–[20]. Given a landscape conductance matrix and the location of the putative cradle area of the crop, we calculate the least-cost distance to all archaeological site locations for which we have dated prehistoric crop remains, following [18]. These distances are then used as a predictor of the arrival date of the crop at those sites. The earliest dates for each area are the most relevant, as these are indicative of the introduction of the crop to that area, while later dates correspond to local expansion of the crop within the area or to a failure to detect earlier crop remains. To focus our analysis on the sites with the oldest crop remains rather than the average age for a given area and to avoid defining discrete areas, we use quantile regression [38]. Quantile regression is used for regression on a quantile (τ), like the median (τ = 0.5). By setting τ to a relatively extreme value (τ>0.75), we increase the influence of the oldest sites in the analysis. Quantile regression is robust in dealing with skewed distributions and outliers, which makes it especially suited to our approach, which precludes checking error distributions and removing outliers. We use the pseudo-R^2^ of quantile regression, R^1^, as the goodness-of-fit [39].

#### Modelling heterozygosity

Dispersal is expected to leave a mark on the diversity within and between populations. During the expansion of humans out of Africa and spread across the world, each time generally small groups split off to occupy new areas, taking with them only a portion of the alleles from their original population. As a result, human populations show a regular decline in heterozygosity from Africa to the southern tip of South America [32]. For crops, a similar effect is to be expected. In grain crops, mostly whole infructescences are selected for seed. This reduces the number of maternal parent plants and hence the effective population size. If seed lots are relatively small, genetic bottlenecks occur. In established traditional farming systems, pollination between fields and seed mixing tend to counteract the resulting loss of alleles and maintain diversity levels [40]–[41]. During range expansion, however, seed lots are taken into new territory, beyond the reach of these diversity-restoring processes. Also, seed quantities during crop expansion may have been limited by several causes. If crop expansion was due to human migration, such migration movements may have been motivated by push factors like marginalization or persecution. If so, migrants may have arrived with few resources, including seeds. If crop expansion was a process of cultural exchange, hunter-gatherers with no agricultural experience or farmers adopting a new crop were the ones who took the crop further into new territory. Therefore, cultivation in these new locales must often have been precarious and experimental in nature, possibly leading to small seed lots and low crop plant survival rates. Therefore, we think it is reasonable to expect a loss of diversity during crop dispersal, resulting in a declining gradient of crop diversity from the origin.

Like for crop remain ages, the least-cost distance from the origin of the crop should therefore be a good predictor for heterozygosity levels. Here, for simplicity, we determine the cost distance to predict heterozygosity from the same landscape model as we use for crop remain ages. This assumes that the loss of diversity due to genetic bottlenecks through each area is proportional to the time it took to cross these areas. This may not be realistic. The intensity of genetic drift is related to population size, which may change over time and among agricultural systems. Also, the number of bottlenecks over a given distance may differ. See below, under *Discussion* *–Next modelling iterations*, for a refinement of this aspect of the approach.

Selection, introgression from wild populations, as well as recent founder effects and subsequent hybridization may all confound the spatial pattern of heterozygosity. However, as long as the pattern is mainly due to the initial wave of dispersal and not to subsequent long-distance gene flow events or introgression from wild relatives, the net effect of these subsequent demographic events will be to *decrease* heterozygosity locally. If this is the case, the upper limit of heterozygosity will be largely determined by the least-cost distance from the crop origin. Heterozygosity levels that fall short of this maximum will have undergone more recent drift or selection. Only the upper limit of heterozygosity contains information about prehistoric crop dispersal. This problem is similar to the one encountered with the crop remain age data, where we are also primarily interested in the highest values of each area. Therefore, the quantile regression approach introduced above can be used for heterozygosity as well.

#### Modelling genetic distances

During dispersal, the genetic divergence between populations is due to the progressive isolation of populations as their trajectories split. The earlier trajectories split, the more genetic divergence is to be expected. On the other hand, populations that share a large part of their trajectory will undergo a common loss of alleles (alleles which may continue on pathways in other directions from the origin) and have a higher degree of common ‘surfing’ alleles, which have emerged at intermediate locations [42]. Both effects will lead to a higher genetic similarity between populations that share a longer trajectory from the origin.

Ramachandran et al. predicted genetic distances between human populations with distances along dispersal routes out of Africa through waypoints [32]. This gave better results than predictions with direct geographic distances between the sampled populations (as in an isolation-by-distance model). The distance via migration waypoints corresponds to the divergent part of the prehistoric migration trajectories of each pair of populations. Hence, genetic distances between human populations reflect their migration history and arguably the same is the case for crops. We extend this approach in two ways: (1) using a grid-based landscape model, as described above, thus obviating the need for discrete waypoints, and (2) taking into account not only the divergent part of trajectories, but also the length of the shared part. Again, we assume that genetic drift during crop dispersal was constant in time.

During crop dispersal, the first varieties to reach a new place will have more probability to be taken further than varieties that arrive there later. Hence, the dispersal route of individual alleles will be close to the shortest (least-cost) path from the origin location to the location of the sampled population. However, there will also be movements of sideward gene flow along the expansion front that will canalize genes towards parallel paths, bringing in a random element. Random walks can be modelled with analytical methods, using the analogy with electrical current, to avoid repeated simulations to determine probabilities [35]–[36]. Random walk distances are useful to model genetic distances in heterogeneous landscapes for gene flow in an equilibrium situation [30]. However, gene flow during range expansion is intermediate between a least-cost path and a random walk. This could be represented with *randomized shortest paths*, which allow varying the degree of constraint to the least-cost path, changing the value of θ [43]. Parameter θ can be varied between 0 (random walk) and ∞ (shortest path). A randomized shortest path is also somewhat longer than the shortest path, but in this context, the correlation between the two types of distances is high and tends to 1 when θ approaches ∞.

**Figure 1:**
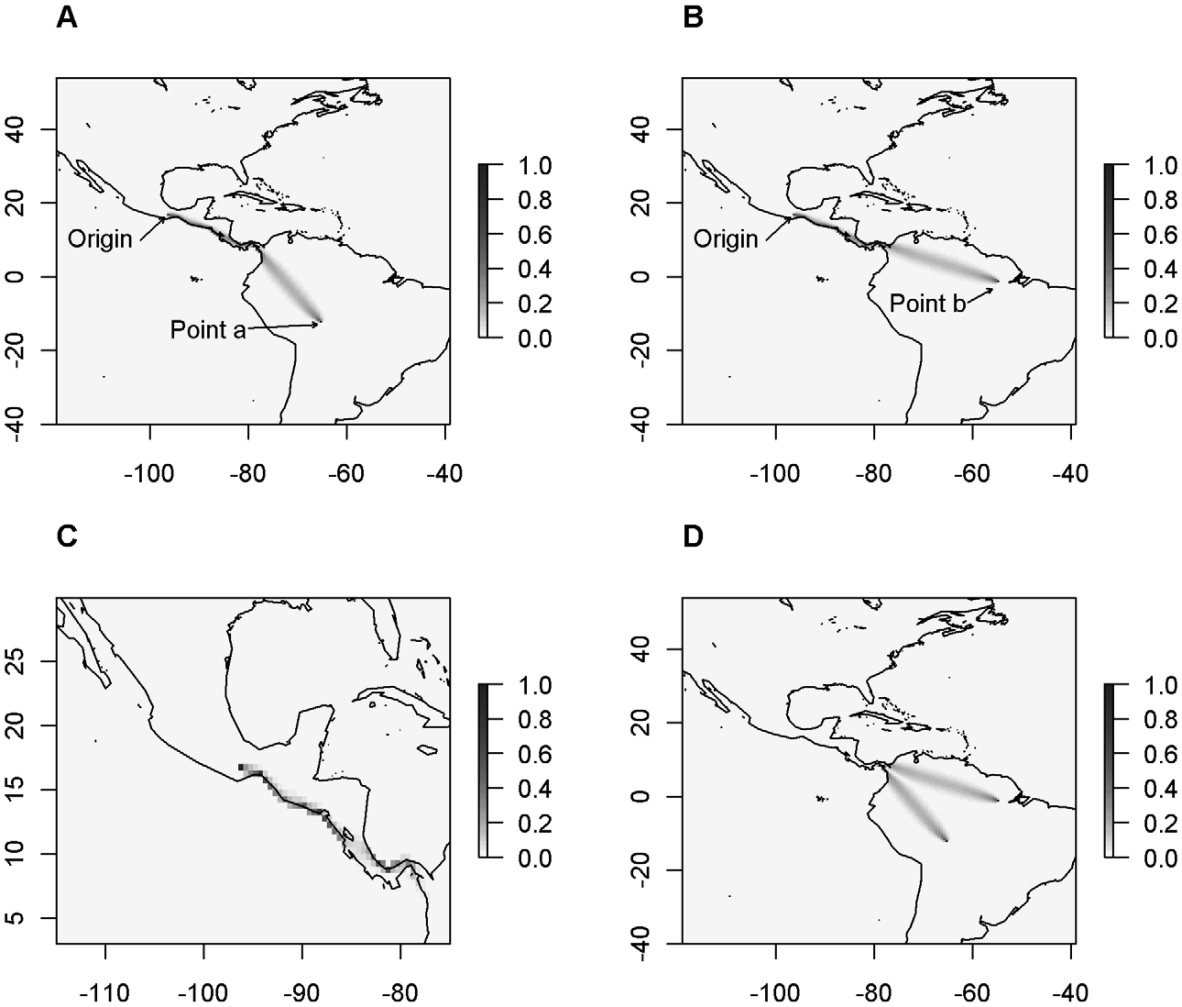
Example of trajectory overlap and divergence calculation for two populations. Both populations are from South America (point locations A and B). The origin of the crop is in Mexico. A. Probability of passage from origin to location a. B. Probability of passage from origin to location b. C. Overlap of the trajectories. D. Divergence of the trajectories.

For a given origin, destination, conductance matrix, and a value for θ, we calculate the *net* number of times of transition over each cell connection, i.e., the number of transitions not reciprocated by transitions in the opposite direction. We take this to correspond to the probability of passage (*P*) of the forward movement of an allele during the wave of expansion. With a given origin, we calculate the matrix *P* for each sample location. Each transition probability matrix *P*_a_ represents the stochastic trajectory from the origin to point a. The probability that two different trajectories (*P*_a_ and *P*_b_) coincide in connections between cells can be calculated by multiplying the two matrices:
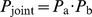
(1)
*P*_joint_ is a matrix with the probabilities of joint passage for each cell connection. Likewise, to determine to what extent connections between cells are part of the divergent part of the trajectories, we calculate the probability that the most probable trajectory crosses a cell connection and the least probable trajectory fails to do so. If this probability exceeds the probability that the least probable trajectory crosses the cell, there is enough asymmetry between the trajectories to consider the cell connection as part of the divergent part of the trajectory:
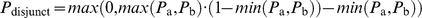
(2)
Figure 1 illustrates these calculations by showing the transition probabilities by cell. We multiply the obtained matrices *P*_joint_ and *P*_disjunct_ with the resistance matrix, R. (Here, we determine R as the reciprocal of the conductance matrix of the landscape model, but see below under *Discussion* *–Next modelling iterations* for an extension to this.) We then sum the values of the whole matrix.
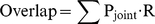
(3)

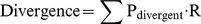
(4)We repeat this procedure for all pairs of sample locations. The two obtained variables, path overlap and path divergence, can be compared with the pairwise genetic distances using regression methods.

#### Fitting the model

Using the computational strategies outlined above, we can derive from the landscape model different distance measures that relate to crop remain age, heterozygosity, and genetic distances. We use multiple-criteria optimization to evaluate how well our model can explain the archaeobotanical *and* genetic observations. Multiple-criteria optimization is an underutilized technique with interesting applications to detect conflicts between model structure and patterns in the data [44].

Multiple goodness-of-fit values are determined by regression of the predictors against the archaeobotanical and genetic data. A genetic algorithm optimizes two or more goodness-of-fit measures through an iterative search of the best parameter values and origin coordinates. The outcome of this optimization is a Pareto front of solutions. Pareto solutions are those for which improvement in one goodness-of-fit dimension can only occur with the worsening of at least one other goodness-of-fit dimension. The shape of the Pareto front gives a good indication of the degree of convergence or conflict between the two datasets, given the model structure. A pointed, convex front (seen from the cloud of possible solutions) is evidence for convergence around the same solution. Inspecting the parameters and origin coordinates of the different solutions can provide insights into the source of the conflict and thus help in improving the model structure.

### Application

#### Computational implementation

The data analysis was done in *R*, a free and open-source computer program and language for data analysis [45]. The examples can be replicated with the script and data provided as Supporting Information. Basic geographic grid manipulation and calculations were done with functions from *R* package (plug-in) *raster* [46]. The creation of conductance matrices from grid data, their manipulation, and the calculation of geographic distance measures were supported by functions in *gdistance* [47]. Package *gdistance* makes use of sparse matrices [48]. Methods to analyze distance matrices and calculate genetic distances are implemented in *gdistanalyst* [49]. We used the *R* package *mco* for the multi-criteria optimization [50], which implements the algorithm NSGA-II [51]. An R script to replicate the procedure is available as Supplementary Information (file S1).

#### Landscape model

We modelled maize dispersal and diversity with a simple landscape model. We used a grid of 0.5 by 0.5 degree resolution, covering the study area. Grid cells were connected in eight directions (queen's case) to form conductance matrices. The area of origin was modelled as a single cell, which could be anywhere on land. The landscape model includes only information about the shape of the landmass to keep our example as simple as possible, but additional variables could be added (see *Discussion*). The landmass grid is included as Supplementary Information (file S2).

The conductance of between-cell connections on land was set to 1. The conductance of major water bodies was modelled with a decay function and a weight relative to the conductance of the landmass (p_1_), following [20]. Conductance decays with the distance away from the coast (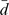, the average distance from the coast of cell i and j) with a constant decay rate (p_2_, the conductance half-value distance). Conductance over water bodies was calculated as
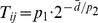
(5)We symmetrically normalized the water body conductance matrix [52]. All conductance values (land and water) were divided by cell-to-cell distances (i.e., distances between cell centres) in order to correct for the differences between diagonal and straight distances between cells and to correct for variations in spherical distances between cells.

#### Archaeobotanical data

We limited the analysis to macrobotanical remains of maize, which at the moment is the only type of archaeological remains of this crop for which there is a somewhat complete coverage of the Americas. Radiocarbon dates were derived from [53] and supplemented with a number of additional dates from other publications (Supplementary Information, file S3). We calibrated the raw dates with OxCal [54], using IntCal04 [54] for the Northern Hemisphere and ShCal04 [56] for the Southern Hemisphere. In the tropics, we took a weighted average of the two median values, the weights depending on the latitude of the sample location. Outside the tropics, we used the corresponding median values. In a small number of cases, uncalibrated dates were not available and published calibrated dates were used directly.

#### Genetic data

We used genetic data from [22] on 193 maize landrace accessions from across the Americas, based on one plant per sample and 99 SSR markers. We calculated the heterozygosity for all samples and used the logarithm of the shared proportion of alleles as the genetic distance between the samples. As we reorganized the data, in order to replicate the procedure, we supply it here as Supplementary Information (files S4 and S5).

#### Model fitting and evaluation

We first optimized the landscape model with (1) the age of the archaeobotanical crop remains and (2) the heterozygosity of contemporary maize samples. For a number of the Pareto solutions obtained, we then evaluated the goodness-of-fit with (3) the genetic distances. We choose this setup in two rounds to reduce computation time and to test the performance of our new path overlap and divergence metrics independently. The path overlap and divergence metrics should predict the genetic distances well if the modelling approach is coherent.

In the first round, the goodness-of-fit was determined with quantile regression, setting τ to 0.8 for both radiocarbon age and heterozygosity. Since the archaeobotanical data were highly unequally spread with an especially high density of observations in Colorado, New Mexico, and Arizona, we weighted each observation by 1/number of observations within a radius of 100 km from that observation (including the observation itself). Hence, an observation with one neighbour within 100 km distance received the weight 0.5, while an isolated observation was weighted as 1.

We optimized with a population of 200 during 60 generations. Further improvements after 200 generations were minimal and solutions showed a regular pattern. We selected for further analysis a subset of nine representative Pareto solutions. We calculated path overlap and divergence based on these solutions for various values of θ. We evaluated the correspondence between path overlap/divergence and genetic distances with linear permutational regression with 999 permutations [57].

## Results

**Figure 2:**
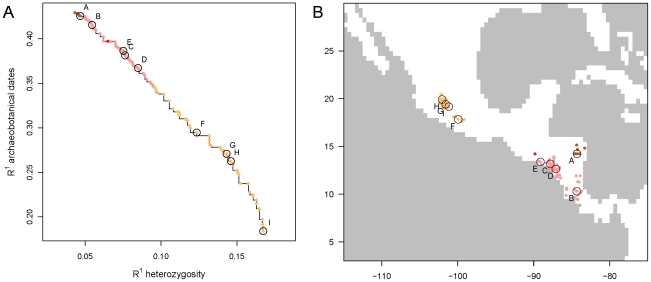
Pareto solutions obtained by fitting the landscape model with archaeobotanical and genetic data. A. Model fits at the Pareto front, with selected solutions labeled from A to I. B. Inferred locations of crop origin, with colors and labels corresponding to those of Figure 2A.

We obtained a set of Pareto solutions that were overall similar. We provide the full set of obtained solutions as Supplementary Information (file S6) and summarize it here (Table 1, Figure 2). In all solutions, the geographical origins of maize fall in Mesoamerica. The absolute R^1^ values for the fit with heterozygosity were low for all solutions, indicating that heterozygosity may be influenced by recent population bottlenecks and selection. The solutions with a higher fit for heterozygosity suggest a more northern origin of maize than those that correspond better to the archaeobotanical data. A strong source of tension between the archaeobotanical and genetic data is the conductance of water bodies, with the genetic data suggesting that water bodies are less conductive than what would be expected from the pace of dispersal according to the archaeobotanical data. The solutions with good archaeological fit have higher half-value distances (p_2_) than those with a good genetic fit. Hence, the main effect of the high weight given to water bodies in the latter solutions is to increase the conductance along the coast.

**Table 1:** Representative selection of Pareto solutions.

Solution	Area of origin		Water bodies		Goodness of fit (R^1^)	
	Longitude	Latitude	Relative weight (p_1_)	Conductance decay half-value distance (km) (p_2_)	Archaeobotanical crop remain ages	Heterozygosity of contemporary maize samples
A	−84.29	14.22	0.23	5310	0.43	0.05
B	−84.34	10.31	0.18	5991	0.42	0.05
C	−87.81	13.17	2.03	3065	0.38	0.08
D	−87.08	12.63	2.39	3018	0.37	0.08
E	−89.11	13.37	2.12	3093	0.39	0.08
F	−99.94	17.83	6.10	2631	0.29	0.12
G	−101.58	19.41	7.51	2569	0.27	0.14
H	−102.06	19.91	8.39	2519	0.26	0.15
I	−101.17	19.17	16.17	2347	0.18	0.17

**Figure 3:**
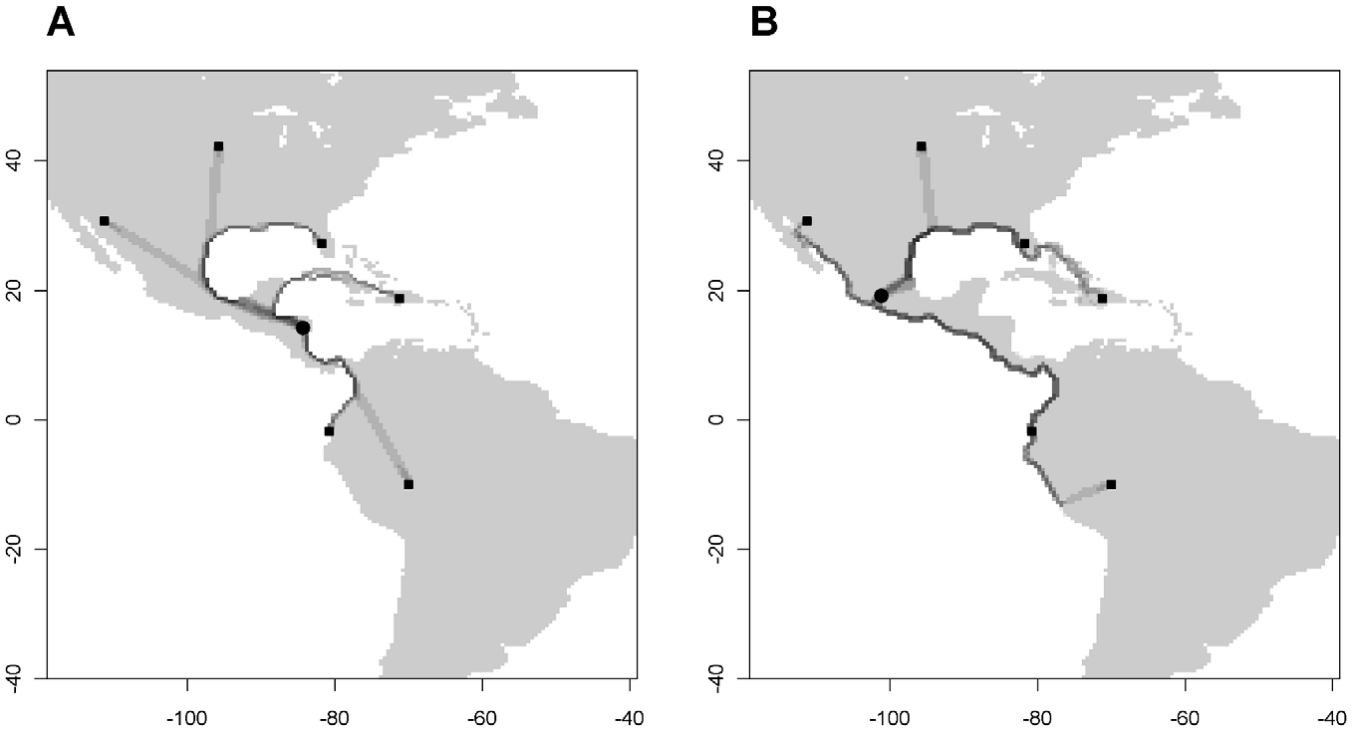
The dispersal routes of maize in the Americas for two contrasting Pareto solutions. Routes determined with randomized shortest paths (θ = 0.2) and logarithmically scaled. A. Routes of dispersal from origin (circle) to six locations (squares) according to solution A. B. Routes of dispersal from origin (circle) to six locations (squares) according to solution I.

**Figure 4:**
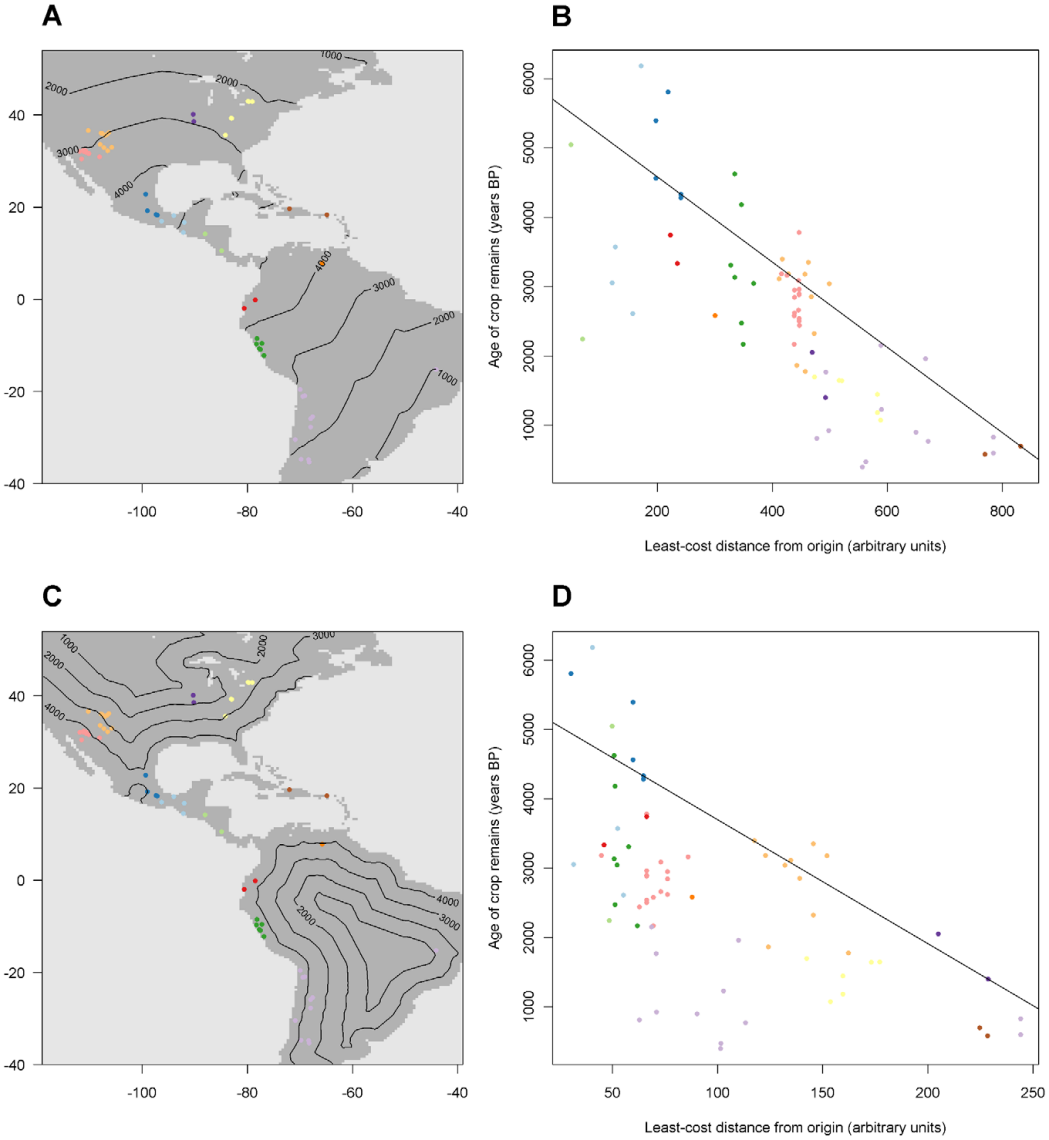
Crop remain age according to two contrasting Pareto solutions. A. Locations of the archaeobotanical observations with modelled isochrons (oldest quintile of macrobotanical remains) for solution A. B. Relation between the age of crop remains and the least-cost distance from the crop origin according to solution A. The colors of the observations correspond to Figure 4A. The line indicates the highest quintile (τ = 0.8) predicted by the model. C. Locations of the archaeobotanical observations with modelled isochrons (oldest quintile of macrobotanical remains) for solution A. D. Relation between the age of crop remains and the least-cost distance from the crop origin according to solution A. The colors of the observations correspond to Figure 4C. The line indicates the highest quintile (τ = 0.8) predicted by the model.

**Figure 5:**
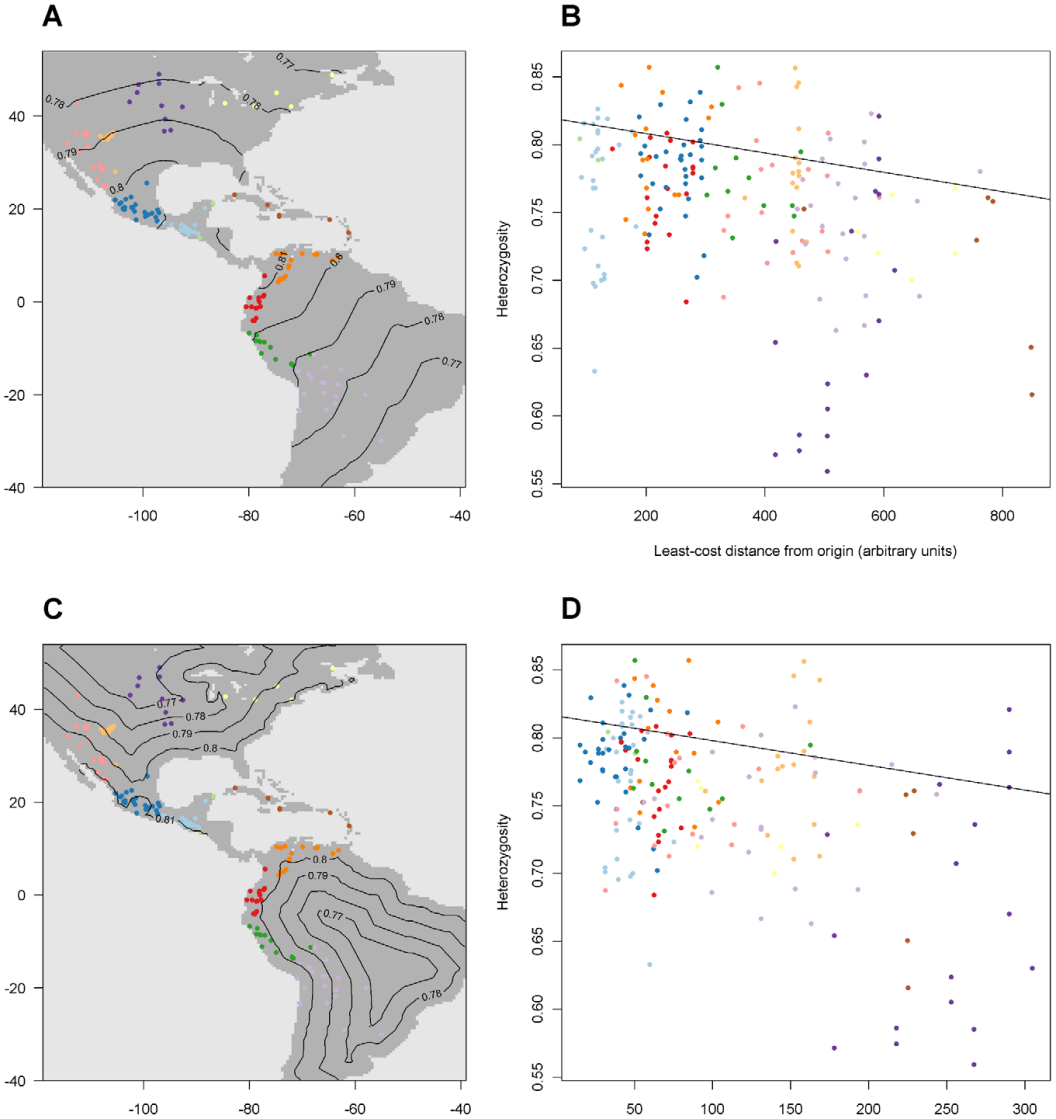
Heterozygosity according to two contrasting Pareto solutions. A. Locations of the genetic observations with isolines indicating modelled heterozygosity values (highest quintile) for solution A. B. Relation between heterozygosity and the least-cost distance for solution A. The colours of the observations correspond to Figure 3A. The line indicates the highest quintile (τ = 0.8) predicted by the model. C. Locations of the genetic observations with isolines indicating modelled heterozygosity values (highest quintile) for solution I. D. Relation between heterozygosity and the least-cost distance for solution I. The colours of the observations correspond to Figure 3C. The line indicates the highest quintile (τ = 0.8) predicted by the model.

Visualizing and comparing the different solutions provides some additional information. In Figures 3, 4 and 5, we compare two solutions (A and I) which gave the best fit for the archaeobotanical and genetic data, respectively (compare with Table 1 and Figure 2). The effect of the difference in conductance of the coast is clearly visible when we map the dispersal routes (Figure 3). While in solution A, there is a slight preference for dispersal along the coast (Figure 3A), in solution I, dispersal avoids non-coastal areas as much as possible (Figure 3B).

Figure 4 compares the archaeological outcomes. The archaeological data are clearly spatially patterned. The fit of solution A is fairly good, although the five data points closest to the origin of solution A are younger than expected (Figure 4B). The major discrepancies of solution I are in areas near the coast, where ages are clearly underestimated.

Figure 5 compares the outcomes for heterozygosity. There is a high degree of variation in heterozygosity values within each geographical group, yet broad spatial trends are evident. Samples with extremely low heterozygosity values are present in parts of North America. A main difference between the two solutions concerns these samples. In solution A, the prediction of heterozygosity of these samples is clearly too high (Figure 5B). In solution I, the prediction differentiates between North American samples that are closer to the coast, which receive higher values, and the more inland samples, which receive lower values. The difference in conductance of water bodies affects the Caribbean samples to a much lesser extent. In both solutions, heterozygosity values for samples in the Caribbean are lower than expected (all are below the regression line).

We used least-squares regression to determine the degree in which our dispersal model explains the variation in genetic distances, for different values of θ (Table 2). Path overlap and non-overlap derived from nine representative Pareto solutions were tested. An R^2^ of 0.36 to 0.38 was found for solutions F, G, H, and I. These are also the solutions with the highest fit for heterozygosity (Table 1). An isolation-by-distance model (using great-circle distances) on the same data gave R^2^ = 0.16. All solutions provided significant predictors of genetic distance (p<0.001). The two variables had the expected sign (negative for path overlap, positive for divergence) in all cases, except for path divergence in solution A and B with θ values of 1.5 and 2. In these cases, the contribution of path divergence was insignificant (0.02<p<0.26).

**Table 2:** Proportion of variance explained (R^2^) by path overlap and divergence variables for different solutions and different values of *θ*.

*θ*	A	B	C	D	E	F	G	H	I
0.01	0.19	0.16	0.16	0.16	0.18	0.26	0.35	0.38	0.38
0.1	0.23	0.15	0.20	0.20	0.22	0.36	0.36	0.36	0.31
0.5	0.27	0.15	0.28	0.27	0.29	0.36	0.34	0.35	0.25
1	0.15	0.12	0.28	0.27	0.30	0.32	0.34	0.33	0.22
1.5	0.06	0.05	0.23	0.24	0.25	0.32	0.33	0.30	0.22
2	0.02	0.04	0.11	0.18	0.11	0.33	0.32	0.28	0.22

C. Locations of the genetic observations with isolines indicating modelled heterozygosity values (highest quintile) for solution I.

D. Relation between heterozygosity and the least-cost distance for solution I. The colours of the observations correspond to Figure 3C. The line indicates the highest quintile (τ = 0.8) predicted by the model.

## Discussion

### Application

With a simple model for maize dispersal, we obtained a set of solutions with similar geographical origins. Multi-criteria assessment revealed the conflict between the archaeological and genetic datasets and gave clues regarding the possible causes for this tension. Different solutions can be inspected visually and compared. This allows us to formulate next steps to iteratively improve the model in a focused way.

One possible explanation of the observed conflict is that, along the coast, maize was transported over large distances, each movement causing a single genetic bottleneck, while over land, spread was more continuous and movements were shorter and alleles were lost each time seeds passed from hand to hand. Hence, differences between the genetic outcomes of dispersal over water versus land may partly explain the observed conflict between the two datasets. However, it seems more likely that the bottleneck observed in North American maize is related to geographical factors that are not taken into account here. The samples with low heterozygosity include Northern flint varieties. Northern flints are known to constitute a genetically very separate group [58]. In this case, selection for climate adaptation seems to have led to a genetic bottleneck that is disproportionate to the time that it took for these varieties to occupy their area.

The results also demonstrate a fairly good predictivity of path overlap and non-overlap metrics for genetic distances. The best of the obtained goodness-of-fit values are reasonable when considering the simplicity of our model and values obtained in similar studies [cf. 59].

### Next modelling iterations

The results show that the speed of dispersal does not correspond linearly to the loss of heterozygosity. Other factors of influence not included in the current model certainly have importance, like climatic factors in the case of the Northern flints. However, adding more variables to the model will affect dispersal speed and the loss of heterozygosity equally and will not break their linear relationship. The discrepancy can only be resolved by relaxing the assumption that genetic drift was constant in time and determining (archaeological) “travel time” and (genetic) “travel cost” separately.

In a next modelling iteration, the model should be expanded to make this possible. Shortest paths from the origin in the landscape model should be used to predict crop remain age. Also, the trajectories from the origin to the genetic samples should be determined on the basis of the landscape model. However, a separate genetic conductance matrix should then used to calculate the cost of these routes in order to predict heterozygosity and genetic distances. For the path overlap and divergence metrics, this means that R (resistance) in formulae 3 and 4 is not determined as the reciprocal of the landscape model, but as the reciprocal of this genetic conductance matrix. Predictors for heterozygosity can also be determined with this same matrix. The parameters to construct the genetic conductance matrix are added to the multiple-criteria optimization. Otherwise, the approach remains the same. This extension of the approach would be a logical next iteration in our modelling exercise.

Next modelling iterations should also make use of additional information to refine the landscape model. For example, ecophysiological crop models [60] could be used to predict the degree in which a crop can grow in a new environment in combination with paleoclimatic reconstructions [cf. 61]. Insights in human demography [62] and mobility (e.g., navigation abilities [21]) could be incorporated as well. Also, the interactions between different crops (as well as livestock) and their spread as single agricultural complexes would be important to consider. The degree of consilience reached by the model, as indicated by the convexity of the Pareto front, can be used to assess model improvement, while cross-validation could be used to guard against overfitting.

Our results make clear that next modelling steps should ideally incorporate the genetic distances directly into model fitting. Genetic distances are often easier to obtain than reliable heterozygosity values and the accuracy of heterozygosity values is often limited by small sample sizes. For inbreeding crops, heterozygosity values are not available or meaningful and only genetic distances can be used. As the repeated computation of path overlap and divergence is very time-consuming in the current implementation, reducing computation time is a priority. Parallel computing approaches can be used at several levels. On the other hand, it has been found that relatively coarse grids still produce relatively accurate results [63].

### Methodological considerations

Choosing appropriate values for τ and θ is an important issue. Values for τ should be determined taking into account the magnitude of error or bias in the data. Even so, the best value τ is difficult to determine beforehand. The best value of θ is also difficult to determine beforehand, although reference values may become available if our approach is applied to various crops and dispersal processes. Sensitivity analyses could be applied to determine the influence of these two parameters.

We used least-squares regression to quantify the variance of the genetic distances the model was able to explain. As with crop remain age and heterozygosity, bias reduction in genetic distances could be achieved using quantile regression (giving emphasis to long genetic distances by setting τ to a low value). However, this would be under the assumption that the bias is mainly due to local divergence, not to posterior long-distance geneflow or introgression from wild relatives. For that reason, the quantile regression approach has limitations when working with contemporary genetic data. Bias reduction may also be achieved in other ways, for instance, by selectively removing outliers and introgressed samples. Also, if genetic data for archaeological crop remains become available, it should become possible to obtain a clearer genetic signal of the first wave of dispersal, which would then help to distinguish it from the changes that occurred after this first wave (local divergence, foreign introductions, and hybridization). For maize, long-distance gene flow after the first wave of dispersal seems to be due to relatively recent (colonial and post-colonial) migration and trade [64].

Above we have modelled the geographical crop origin as a point location, but a crop origin may also be modelled as an area with a certain extent to assess the possibility of a ‘protracted’ domestication process, in which a crop evolves during a long period, perhaps several thousands of years, in an extended region, before spreading to other areas [15]. The approach could also be refined by adding conductance matrices focusing on long-distance connections in certain areas. For instance, between (groups of) islands, the spread of crops may have taken place in less predictable ways. Transport over sea can be modelled with separate conductance matrices, each connecting a pair of non-adjacent cells (or a group of them). Each conductance matrix then receives a separate weight parameter in the multi-criteria optimization. Future work should also extend the approach to address crops with more complex trajectories, involving multiple origins and hybridization between populations domesticated in different areas, which would require further methodological development.

### Contribution

The main methodological innovations in our approach are (1) the use of parameterized landscape conductance matrices to construct landscape models, (2) the use of quantile regression to reduce noise in radiocarbon dates of crop remains and heterozygosity values, (3) the use of multi-criteria optimization for simultaneous model assessment, and (4) the introduction of path overlap and divergence as measures to predict genetic distances. An important strength of our approach is that parameterization can be done in a fully automated way. There is no need to force routes through certain waypoints, to determine landscape conductance *a priori* or through piecemeal trial-and-error or to devise complex methods to select the earliest data points, as was done in studies with similar aims [16]–[20], [31], [65]. Different types of geographic distances predictive of archaeobotanical and genetic measures can be derived directly from our landscape model.

Models with more variables and parameters can be evaluated with the presented methods and should lead to an increase of one or more goodness-of-fit values while not deteriorating the other values. The possibility to incrementally move from a simple initial model to more complex models, as comprehension of the processes studied increases, is crucial to successful modelling. It provides for a modelling approach that is driven by an understanding of the dominant processes supported by the data, while avoiding unnecessary details and computational effort. As the resulting models would be based on a representation of the underlying geographical processes, they could be used to predict levels of biodiversity in unsampled locations and lead to applications in genetic resources management.

## Supporting Information

File S1 R script. Script in R which replicates the complete analysis presented in the manuscript. Instructions to run this script: 1. Put all the documents in a single folder (this becomes your working directory) 2. Install the necessary packages in R (see first part of the script) 3. Define the working directory in the script (setwd(“C:/…/”), as indicated in the script) 4. Run the script in R
(0.02 MB TXT)

File S2 Sea mask. Geo-data in ASCII format: location of water bodies and land, 0.5×0.5 degree resolution.
(0.06 MB TXT)

File S3 Maize archaeobotanical database. Excel file with radiocarbon data and coordinates used in the analysis.
(0.14 MB XLS)

File S4 Maize SSR data. Excel file with the genetic (SSR) data used in the analysis, from ref. 22. The IDs were corrected to match IDs of file 5.
(3.48 MB XLS)

File S5 Plant samples maize SSR data. Excel file with further information on the samples used in the SSR analysis of File 4, from ref. 22. The IDs were corrected to match IDs of file 4.
(0.13 MB XLS)

File S6 Pareto solutions. Complete set of Pareto solutions generated by the analysis.
(0.02 MB CSV)
